# Wound Complications Following Resection of Adductor Compartment Tumours

**DOI:** 10.1080/13577140120099191

**Published:** 2001-12

**Authors:** Melvin F. Grainger, Robert J. Grimer, Simon R. Carter, Roger M. Tillman

**Affiliations:** 1 Royal Orthopaedic Hospital Birmingham UK; 2 25 Baldwin Road Kings Norton Birmingham B30 3LB UK

## Abstract

*Purpose* Limb salvage surgery of soft tissue sarcomas is associated with both a risk of local recurrence and wound complications. Although the lower limb appears to be at greater risk of wound-related morbidity, few studies separate anatomical compartments. We believe that the adductor compartment of the thigh has a particularly high rate of complications and so performed a retrospective analysis of all soft tissue sarcomas arising in this region undergoing limb salvage.

*Patients* Patients with intermediate and high grade adductor compartment tumours were identified from our database and the case notes were reviewed for patient, tumour, surgical and wound variables, identifying those with wound complications both before and after discharge.

*Results* Of 49 patients who underwent limb salvage surgery, 22 (42.9%) developed complications. Twelve patients (24.5%) required further surgery prior to wound healing and 10 patients had delays in post-operative radiotherapy. There were significant differences in the rates of preceding surgery, open biopsy performed at other centres and previous radiotherapy to this region between the complicated and uncomplicated groups.

*Discussion* The management of these difficult tumours carries a high rate of wound complications and requires careful planning prior to tissue biopsy. Open biopsies should be performed by the tumour surgeon to allow easy inclusion of this site in the definitive procedure. In previously irradiated or operated limbs, alternative strategies for wound management may need to be considered.


*Sarcoma* (2001) **5**, 203–207

**ORIGINAL ARTICLE**

### Wound complications following resection of adductor compartment tumours

MELVIN F. GRAINGER, ROBERT J. GRIMER, SIMON R. CARTER & ROGER M. TILLMAN

*Royal Orthopaedic Hospital, Birmingham, UK*

**Abstract**

*Purpose* Limb salvage surgery of soft tissue sarcomas is associated with both a risk of local recurrence and wound complications. Although the lower limb appears to be at greater risk of wound-related morbidity, few studies separate anatomical compartments. We believe that the adductor compartment of the thigh has a particularly high rate of complications and so performed a retrospective analysis of all soft tissue sarcomas arising in this region undergoing limb salvage.

*Patients* Patients with intermediate and high grade adductor compartment tumours were identified from our database and the case notes were reviewed for patient, tumour, surgical and wound variables, identifying those with wound complications both before and after discharge.

*Results* Of 49 patients who underwent limb salvage surgery, 22 (42.9%) developed complications. Twelve patients (24.5%) required further surgery prior to wound healing and 10 patients had delays in post-operative radiotherapy. There were significant differences in the rates of preceding surgery, open biopsy performed at other centres and previous radiotherapy to this region between the complicated and uncomplicated groups.

*Discussion* The management of these difficult tumours carries a high rate of wound complications and requires careful planning prior to tissue biopsy. Open biopsies should be performed by the tumour surgeon to allow easy inclusion of this site in the definitive procedure. In previously irradiated or operated limbs, alternative strategies for wound management may need to be considered.

### Introduction

In patients with extremity soft tissue sarcomas (STS), the distress caused by the diagnosis may be exacerbated when surgical treatment causes further morbidity resulting in prolonged hospitalisation, further surgical procedures, delay in adjuvant treatment and possibly the loss of the limb. Studies into complications following resection of musculoskeletal tumours reveal rates ranging from 8 to 51%.[1–3] The rate is influenced by the definition of a complication, inclusion of multiple anatomical sites and type of surgery included in the data.[4,5] Others include tumours that have been irradiated either pre-operatively or in the immediate post-operative period.[2,6] Our experience is that of all anatomical sites the adductor compartment of the thigh is particularly prone to wound healing complications. We therefore performed a retrospective analysis of our patients to identify all patients with wound complications, to try and identify possible factors that may identify the 'at risk wound' and allow us to formulate management strategies that will reduce the incidence of such problems.

### Patients and methods

Patients referred to our unit between January 1989 and April 1999 with adductor compartment STS beneath the deep fascia were identified from our database. All patients undergoing limb salvage surgery with Trojani grade 2 or 3 tumours, regardless of previous treatment were included. Grade 1 lesions were not included as these were largely shelled out low grade liposarcomas. Lesions arising in bone and extending in to the adductor compartment were excluded. Forty-eight patients were identified by these criteria of which 12 had Trojani grade 2 and 39 Trojani grade 3 tumours. One patient was treated for a liposarcoma but on later review of the histology it was recognized to be a myxoma. This patient has been included since surgical management was as for the other higher grade lesions. Individual case notes were reviewed and data regarding patient, tumour, surgery and wound details were recorded.

Needle biopsy was usually performed in clinic to give a tissue diagnosis, although open biopsy was performed if the initial biopsy was inconclusive. Perioperative antibiotic prophylaxis depended on the preference of the

Correspondence to: Melvin F. Grainger, 25 Baldwin Road, Kings Norton, Birmingham, B30 3LB.
E-mail: mel.deb@the-graingers.fsnet.co.uk. Fax: 0121 685 4146

1357–714X print/1369–1643 online/01/040203–05 © 2001 Taylor & Francis Ltd
DOI: 10.1080/13577140120099191

operating surgeon. Only in three patients (6.1%) was no prophylaxis used. The commonest regimes were a single dose of cefuroxime 1.5 g alone (12 patients) or combined with metronidazole 500 mg (12 patients). Thromboprophylaxis was not routinely used unless there was considered to be a specific indication. Wide excision of the tumour and biopsy tract was performed, in most cases resulting in adductor compartmentectomy. In four cases the femoral vessels were considered to be involved and required resection and reconstruction. Adjuvant therapies were performed at other centres and follow up was shared with these

Defining a wound complication poses difficulties in that a single standard is difficult to apply. A complication requiring further surgical treatment represents a major complication in many series[1,2,7–9] and has been adopted here. A moderate wound complication was one associated with a purulent discharge, or wound dehiscence of greater than two centimetres.[1,2] As with other series, erythema, seromas without other complications and haematomas have been regarded as minor complications and have not been included. Delay in adjuvant therapy does not represent a wound complication but has been recorded where this was due to a wound-related problem.

Patients were identified as having no complications, or those with major or moderate complications satisfying one of the above criteria. Differences between the groups were assessed using Students *t*-test and $\chi^2$ where appropriate.

### Results

Of the 49 patients, 31 were male and 18 female with a mean age of 60.3 years (12–89 years). Only two patients were diabetic. Wound complications developed in 21 patients (42.9%) (Table 1). Eight patients had complications prior to discharge, with 13 developing complications subsequently. Twelve patients required surgical debridement of their wounds (major complications) requiring a total of 23 procedures. Wound healing was by secondary intention in eight patients, secondary suturing (three patients) and split skin graft (one patient). Nine of these 12 were re-admitted for debridement, with a mean length of stay of 22.6 days in those where readmission duration was known. Of the nine patients with moderate complication, three patients developed complications prior to discharge, four required admission for wound care and antibiotics and two were managed in the community with wound care and oral antibiotics. Culture results were available in 17 patients with no specific pathogen identified in two. The culture results were unavailable in a further four cases treated at other institutions. Coliforms were the commonest pathogen (eight patients) with *Staphylococcus aureus* in five patients, *Pseudomonas* (two patients), *Klebsiella* (two patients) and *Citrobacter*, *Cinetrobacter* and *Streptococcus* in one patient each. Five patients grew two organisms. Radiotherapy was delayed in 10 patients (three moderate, seven major complications). In three patients radiotherapy was not planned and one was considered too frail. The mean delay in treatment was 2.5 months. In two patients, local recurrence occurred before radiotherapy could be given because of wound complications, one subsequently requiring hindquarter amputation. One wound had yet to heal at 26 months after the initial debridement and one patient was not followed up due to frailty.

**Table 1**. *Management of patients with wound complications*

| Patient no. | Complication type | Prolonged stay or readmitted? | Wound healing | Healed by (months) | Radiotherapy delay (months) | Organism |
|---|---|---|---|---|---|---|
| 1 | Major | Yes | Skin graft | 4 | 3 | *E coli*, *Staph. aureus* |
| 2 | Major | Yes | Primary | 3 | 1 | Cinetobacter |
| 3 | Major | Yes | Secondary | 2 | na | Enterococcus |
| 4 | Major | Yes | Primary | 2 | Not indicated | *Klebsiella*, *E. coli* |
| 5 | Major | Yes | Secondary | Not available | Not indicated | Not available |
| 6 | Major | Yes | Secondary | Not healed | Hindquarter | E. coli |
| 7 | Major | Yes | Primary | 3 | 2 | Citrobacter |
| 8 | Major | Yes | Secondary | 4 | 1 | Klebsiella |
| 9 | Major | Yes | Secondary | 1 | 1 | *E. coli*, *Staph. aureus* |
| 10 | Major | Yes | Secondary | 10 | 10 | Not available |
| 11 | Major | Yes | Secondary | Not healed | N | *E. coli*, *Staph. aureus* |
| 12 | Major | Yes | Secondary | 7 | N | No pathogens identified |
| 13 | Moderate | Yes | Secondary | 1 | N | Not available |
| 14 | Moderate | Yes | Secondary | 1 | 2 | Pseudomonas |
| 15 | Moderate | Yes | Secondary | 3 | 2 | *Staph. aureus*, *Pseudomonas* |
| 16 | Moderate | Yes | Secondary | 4 | 3 | Not available |
| 17 | Moderate | No | Secondary | 2 | N | *Staph. aureus*, *Pseudomonas* |
| 18 | Moderate | No | Secondary | 3 | N | E. coli |
| 19 | Moderate | Yes | Secondary | 1 | N | No pathogens identified |
| 20 | Moderate | Yes | Secondary | No review | Not fit | Coliforms |
| 21 | Moderate | Yes | Secondary | 2 | N | *Streptococcus* G |

Comparison of the results between the complicated and uncomplicated groups is shown in Table 2. There was no significant difference between gender, age, tumour volume, use of wound drain, duration of drainage or drainage volume. The rate of infection was no greater in patients over 60 years (14/21 complicated, 17/28 uncomplicated). The mean preoperative haemoglobin concentration was lower in the complicated group (13.3 g/dl uncomplicated, 12.3 g/dl complicated, $P = 0.05$), although there was no significant difference in the postoperative haemoglobin concentrations or transfusion requirements perioperatively. There was no difference in antibiotic prophylaxis with 14/28 uncomplicated wounds and 11/21 uncomplicated wounds receiving a single dose of cefuroxime and/or metronidazole at the time of induction. Seven patients in each group received antibiotics for over 24 h. The groups showed similar rates use of non-steroidal anti-inflammatory drugs (NSAIDs) and smoking perioperatively. All but two patients had primary wound closure with either subcuticular PDS (polydioxanone) or skin staples and the remaining wounds were closed with split skin grafts. The method of closure was unrelated to outcome. One patient in the complicated group and three in the uncomplicated groups had resection and grafting of the femoral vessels as part of the procedure.

There was a significant difference between the two groups regarding previous treatment. Three patients with complicated wounds had received prior radiotherapy compared with none of the uncomplicated wounds ($P$=0.04). Two had undergone previous excision with postoperative radiotherapy; the other patient received palliative radiotherapy prior to tissue diagnosis for a presumed metastasis. The rate of previous surgery including open biopsy elsewhere was also greater in the complicated group. There were 12/18 (66.6%) wound complications for patients who had initial treatment elsewhere compared to 10/31 (32.3%) whose treatment was solely at our institution ($P = 0.02$). However, if the three patients receiving radiotherapy previously were excluded, this just failed to reach significant levels (9/15 prior open procedure, 10/31 no prior open procedure, $P = 0.07$). There was no significant difference between complication rates between open and needle biopsy performed at our institution.

Comparison of those with major complications and those with moderate complications shown only a lower mean age in those not requiring further surgery for the wound (53.8 vs. 70.2 years, $P = 0.02$). There were no other significant differences as regards any of the other variables.

### Discussion

The aim of limb salvage surgery is to preserve function without compromising oncological principles. Poor wound healing can compromise limb function and ultimately result in amputation.[3,5,7,10–12] The resection of tumours with clear margins creates large wounds that need to have healed before the procedure can be said to have been successful. To our knowledge, this review represents the largest study of the wound complication rate following resection of a STS from a single anatomical compartment. Previous studies into infection rates following STS resection indicate that the lower limb is more frequently affected,[4,6] but few studies separate adductor compartment from 'groin' or 'proximal thigh'.[7] It is not justifiable to assume that anatomical compartments will behave in similar fashions, given they may differ in vascular anatomy and lymphatic drainage. In our experience, adductor compartment of the thigh fares particularly badly.

Our total complication rate of 42.9% is similar to other series,[4,6,7] but these include seroma or haematoma in their data.[1,2,4,6,7] Furthermore studies with rates in excess of 40% also include large numbers

**Table 2.** *Comparison of complicated and uncomplicated wounds*

| | | Uncomplicated wounds ($n$=28) | Complicated wounds ($n$=21) | $P$ value |
|---|---|---|---|---|
| Mean age (years) | | 59.9 | 60.9 | ns |
| Margins | intralesional | 6 | 6 | |
| | marginal | 22 | 15 | ns |
| Mean tumour volume (ml) | | 754 | 820 | ns |
| Biopsy Type | trucut | 23 | 8 | ns |
| | open | 5 | 4 | |
| Previous surgery | | 6 | 12 | $P = 0.01$ |
| Previous radiotherapy | | 0 | 3 | $P < 0.01$ |
| Mean drainage volume (ml) | | 1054 | 956 | ns |
| Mean duration of drainage (days) | | 5.6 | 5.2 | ns |
| Mean preoperative Hb (g/dl) | | 13.3 | 12.3 | $P = 0.05$ |
| NSAID used | | 13 | 11 | ns |
| Smoker | | 7 | 6 | ns |
| Closure method | subcuticular PDS | 22 | 16 | |
| | skin staples | 5 | 4 | ns |
| | split skin graft | 1 | 1 | |

of patients undergoing pre-operative or immediate postoperative radiotherapy which has been shown to influence complication rates.[1,8,9] Excluding those with previous radiotherapy, our wound complication rate remains high at 39.1%. A potential weakness in our study is the inclusion of infections reported from other hospitals. In these cases the complication was considered clinically significant enough to require a change in management or additional treatment and so deemed worthy of inclusion. Twelve patients required further surgical treatment for wound complications (57.1% of complicated wounds, 24.5% of all wounds). This represents a higher complication rate than those series that have used further surgery for wound complications as an endpoint.[4,6,8,9] Using surgery as an endpoint however would have excluded a significant group of patients who had an alteration in their adjuvant treatment or required readmission.

We have included only Trojani grade 2 and 3 tumours as the surgical resection of these is different to the atypical lipoma (grade 1 liposarcoma) which form the vast majority of grade 1 tumours in our experience found in this region. These are usually shelled out with no resection of surrounding muscle and represent a much smaller procedure.

The relationship between factors involved in wound healing is complex, and variables not considered in this study may also play a role.[13] We found no significant relationship between age, gender, smoking, non-steroidal anti-inflammatory drugs or tumour grade, although these have previously been linked with less favourable outcomes,[1,4,6,7,8,14,15] Duration of wound drainage and total volume drained was not associated with outcome although has been previously.[5,6] The low number of diabetics does not allow us to comment on this as a risk factor.

Tumour volume contrary to previous reports was not significant,[1] although the total volume of tissue resected may be more relevant reflecting greater dissection, larger potential dead space and larger skin flaps with relatively ischaemic margins. We have found only one study where vessel ligation has been included in analysis of wound outcome. Data regarding ligation of profunda femoris artery was incomplete but was significantly associated with complications on univariate analysis.[8] We were unable to obtain sufficient operative details to comment on this ourselves.

The majority of the patients seen at our centre had needle biopsy in the outpatient clinic. This has a high degree of accuracy[16] and low infection rate, whereas incisional biopsy requires admission, anaesthesia and has been associated with infection rates up to 17%.[17] Open biopsy at our institution was not related to outcome, open biopsy or excision at other institutions was significantly more common in complicated wounds. This may reflect unrecognised infection in recent surgical wounds, the presence of relatively ischaemic areas in previous excisions or the generation of larger than necessary skin flaps caused by the suboptimal positioning of biopsy wounds. In two cases the biopsy wound was infected pre-operatively. Each had been performed at another institution and despite prolonged courses of parenteral antibiotics both developed wound infections requiring serial debridements. Pre-existing infection in surgical wounds either requires eradication prior to surgery or alternate strategies for wound closure. Although antibiotic prophylaxis is recognised as beneficial in other clean orthopaedic procedures, the lack of standardisation in our series does not allow us to draw any sensible conclusions. The longer courses did not appear to protect against wound infection. The introduction of vascularised tissue into the wound has been associated with better wound healing,[10,18] but this incurs potential donor site morbidity

Limb salvage surgery for adductor compartment tumours is clearly not without risk and full planning with radiotherapists, medical oncologists, tumour surgeons and plastic surgeons is essential. Although no limbs were lost as an immediate result of wound problems, two patients developed local recurrence before radiotherapy could be administered because of their wounds. One subsequently underwent hindquarter amputation and the other received palliative treatment 10 months from surgery having developed pulmonary metastases in the intervening period.

The principle of the surgeon who is to perform the definitive surgery performing the biopsy is understood so that placement of biopsy scars will not compromise later excision. We would suggest that needle biopsy is performed where possible and if open biopsy is required, it is undertaken by the tumour surgeon. Pre-operative attention to factors such as nutrition, diabetic control, smoking habits, as well as careful handling of tissue intraoperatively and attention to haemostasis are essential. Prophylactic antibiotics will doubtless continue to be used but the rationale for this is based on data for other procedures. Closure of the wound following tumour excision is fraught with potential problems. Resections may expose neurovascular structures or bone, which require coverage but primary wound closure is associated with high complication rates. Consideration should be given to the available options of coverage, especially where there are preoperative factors that are associated with the potential for wound complications.

### References

1. Arbeit JM, Hilaris BS, Brennan MF. Wound complications in the multimodality treatment of extremity and superficial truncal sarcomas *J Clin Oncol* 1987; 5(3): 480–8.
2. Ormsby MV, Hilaris BS, Nori D, Brennan MF. Wound complications of adjuvant radiation therapy in patients with soft tissue sarcomas. *Ann Surg* 1989; 210(1): 93–9.

3. Quill G, Gitelis S, Morton T, Piasecki P. Complications associated with limb salvage for extremity sarcomas and their management. *CORR* 1990; 260: 242–50.
4. Saddegh MK, Bauer HCF. Wound complication in surgery of soft tissue sarcoma. *CORR* 1993; 289: 247–53.
5. Chmell MJ, Schwartz HS. Analysis of variables affecting wound healing after musculoskeletal sarcoma resections. *J Surg Oncol* 1996; 61: 185–9.
6. Skibber JM, Lotze MT, Seipp CA *et al.* Limb sparing surgery for soft tissue sarcomas: wound related morbidity in patients undergoing wide local resection. *Surgery* 1987 102(3): 447–52.
7. Bujko K, Suit HD, Springfield DS, Convery K. Wound healing after preoperative radiation for sarcoma of soft tissues. *Surg Obstet Gynaecol* 1997; 176: 124–34.
8. Peat BG, Bell RS, Davis A *et al.* Wound healing complications after soft tissue sarcoma surgery. *Plastic Reconstr Surg* 1994; 93(5): 980–7.
9. Cheng EY, Dusenbery KE, Winters MR, Thompson RC. Soft tissue sarcomas: preoperative versus postoperative radiotherapy. *J Surg Oncol* 1996; 61: 90–9.
10. Barwick WJ, Golgberg JA, Scully SP, Harrelson JM. Vascularized tissue transfer for closure of irradiated wounds after soft tissue sarcoma resection. *Ann Surg* 1992; 216(5): 591–5.
11. Bell RS, O'Sullivan B, Davis A *et al.* Functional outcome in patients treated with surgery and irradiation for soft tissue tumours. *J Surg Oncol* 1991; 48: 224–31.
12. Simon MA, Aschliman MA, Thomas N, Mankin HJ. Limb salvage versus amputation for osteosarcoma of the distal end of the femur. *J Bone Joint Surg* 1986; 68A: 1331–7.
13. Hunt TK, Hopf HW. Wound healing and infection. *Surg Clin* 1997; 77(3): 587–606.
14. Haws MJ, Kucan JO, Roth AC, Suchy H, Brown RE. The effects of chronic ketoralac tromethamine (toradol) on wound healing. *Ann Plast Surg* 1996; 37(2): 147–51.
15. Quirinia A, Viidik A. Diclofenac and indomethacin influence the healing of normal and ischaemic incisional wounds in skin. *Scand J Plast Reconstr Hand Surg* 1997; 31: 213–9.
16. Pitcher ME, Thomas JM. Management of soft tissue sarcoma. *Br J Surg* 1994; 81: 1136–9.
17. Mankin HJ, Lange TA, Spanier SS. The hazards of biopsy in patients with malignant primary bone and soft tissue tumours. *J Bone Joint Surg* 1982; 64A: 1121–7.
18. Cordeiro PG, Neves RI, Hidalgo DA. The role of free tissue transfer following oncological resection in the lower extremity. *Ann Plast Surg* 1994; 33(1): 9–16.
